# Language, culture, and task shifting – an emerging challenge for global mental health

**DOI:** 10.3402/gha.v7.23433

**Published:** 2014-02-27

**Authors:** Leslie Swartz, Sanja Kilian, Justus Twesigye, Dzifa Attah, Bonginkosi Chiliza

**Affiliations:** 1 Alan J Flisher Centre for Public Mental Health, Department of Psychology, Stellenbosch University, South Africa; 2 Department of Psychiatry, Stellenbosch University, South Africa

**Keywords:** mental health, interpreter services, language, task shifting, low income

## Abstract

Language is at the heart of mental health care. Many high-income countries have sophisticated interpreter services, but in low- and middle-income countries there are not sufficient professional services, let alone interpreter services, and task shifting is used. In this article, we discuss this neglected issue in the context of low- and middle-income countries, where task shifting has been suggested as a solution to the problem of scarce mental health resources. The large diversity of languages in low- and middle-income countries, exacerbated by wide-scale migration, has implications for the scale-up of services. We suggest that it would be useful for those who are working innovatively to develop locally delivered mental health programmes in low- and middle-income countries to explore and report on issues of language and how these have been addressed. We need to know more about local challenges, but also about local solutions which seem to work, and for this we need more information from the field than is currently available.

It is well established that language and communication form the basis of good mental health care. It is through language that symptoms are expressed, and language forms the medium through which many aspects of treatment are delivered. There is evidence for the effectiveness of many forms of psychotherapeutic interventions for a range of disorders, and these interventions depend centrally on there being a basis for understanding between clinician and service user (1). Even where treatments are largely biological, as in psychopharmacology, in order for service users to adhere to treatment regimens they have to understand them; this understanding is communicated through language, whether spoken, written, or in another form (2).

With increasing diversity in higher-income countries through migration and large numbers of refugees, a substantial and sophisticated literature has been developed focussing on how best to deal with service users who do not speak the same languages as clinicians. Health services in wealthier countries offer a range of interpreter services to service users from linguistically diverse backgrounds, and mental health interpreting is recognised as an important component of appropriate and accessible care (3). There are various models for these services, ranging from professional interpreting services to community interpreting approaches (the use of trained community members to undertake interpreting as opposed to professionally trained interpreters), but all require investments over and above the usual resources required for care when clinicians and service users speak the same language (2, 4). Quite apart from direct financial costs of interpreter services, there are investments of additional time and infrastructure to accommodate such services, and it is not surprising that the bulk of what is known about interpreting and increasing language access comes from studies in high-income countries (5).

At the other end of the mental health care spectrum, the field of global mental health is acutely concerned with the lack of resources for mental health care in low- and middle-income countries (6). Where mental health professionals are few (and this is the norm rather than the exception for most countries in the world), there is increasing interest in shifting the tasks of professionals to less qualified cadres of workers, thereby making maximal use of nurses, social workers, community health workers and lay personnel in the delivery of care (6, 7). The primary argument for advocating task shifting is that of resources – there are nowhere near as many highly qualified mental health care personnel as would be needed to meet the care burden – but it is also sometimes argued that lower-level workers who deliver mental health care in resource constrained environments may provide even better care than do highly trained professionals. A core reason for this is that community members trained to deliver mental health services are more likely to share cultural, linguistic, and social backgrounds with those receiving care than are professionals (8). Cultural competence in mental health care provision is highly valued, and an aspect of the cultural strengths of lay health workers may be their being native speakers of the languages spoken by mental health service users, whereas clinicians are less likely to be.

In this respect, the position of the lower level or lay health worker in a system using task shifting is analogous in some ways to that of the interpreter (especially the community interpreter) in a mental health system in a higher-income context. Both are commonly positioned as cultural brokers, seen to function as important bridges between the world of professional health care and local understandings (9, 10). The literature on interpreters, and on community interpreters in particular, also describes some of the complexities of this in-between positioning. Interpreters may be erroneously assumed to be experts on the cultural and linguistic worlds of their clients, and be asked to make complex judgements about the cultural acceptability of experiences service users may have. It is not uncommon for interpreters to be asked whether what would be regarded as hallucinations or delusions in western nosologies may in fact be culturally appropriate expressions of distress in the cultural world of health service users (11). Quite apart from the fact that no single person can speak on behalf of a whole cultural group, interpreters may not come from the same social groups as service users even if they are native speakers of the same languages – there are vast differences within language groups regarding beliefs about and experiences of distress and healing (one has only to think of the diversity of monolingual English speakers to realise that this is the case) (12). In global mental health practice, similarly, there may be vast cultural differences between lay health workers and some of those with whom they work.

It is understandable why people concerned about appropriate and accessible service provision would wish that interpreters and lay health workers could be seamless bridges, linguistically and culturally, between western-trained clinicians and diverse populations. The idea that languages and cultures are neatly separable and that cultural and linguistic brokerage is a simple matter of translating from one world-view into another is associated with an outdated and simplistic view of cultures as static entities, and with a lack of appreciation of the complex power dynamics at stake for any person in a brokerage position (13). It is easy to think of lay health workers and interpreters as service user advocates, for example, but where there are large imbalances of power, and especially where low-paid interpreters and lay health workers with no job security are dependent on funding from health systems, they may well feel constrained to behave in ways which they believe may be most acceptable to the professionals who use their services, thereby protecting their own future work (14).

A further consequence of dividing the world neatly into the world of ‘western’ mental health practice and local communities is that this may obscure the linguistic and cultural diversity of low- and middle-income countries.

Table 1 lists the 20 poorest countries in the world, according to World Bank figures, and the figures for psychiatrists per population support the view that task shifting must be an important strategy in these countries – it is not possible for a single psychiatrist to provide direct services for a million people, for example. But the third column in the table gives an indication of the linguistic diversity of these countries – 90% of them have more than 10 local languages and 70% have more than 20. To add to this complexity, a prominent feature of low- and middle-income countries is internal and international migration due to wars, sectarian violence, and climate change, and for economic reasons, to name a few causes.

**Table 1:** Availability of psychiatrists and number of languages spoken in the 20 poorest countries in the world

Twenty poorest countries in the world by GDP (15)	Psychiatrists per 100,000 population (16)	Number of languages spoken (17)
Democratic Republic of Congo	0.11	215
Liberia	0.02	30
Zimbabwe	0.06	19
Burundi	0.01	3
Eritrea	0.06	12
Central African Republic	0.02	71
Niger	0.03	21
Sierra Leone	0.02	24
Malawi	0.01	16
Togo	0.02	39
Madagascar	no data	17
Afghanistan	0.01	48
Guinea	0.03	34
Mozambique	0.04	43
Ethiopia	0.02	85
Mali	0.04	56
Guinea-Bissau	0	21
Comoros	0.14	6
Haiti	0.2	3
Uganda	0.08	43

Figure 1 shows the latest available figures for African refugees according to UNHCR. Apart from the United Kingdom, South Africa, which is a middle-income African country, has the highest official number of African refugees of any country in the world. In addition, it is estimated that 3 million Zimbabweans, and possibly more, are living in South Africa, many of them illegally, and undocumented migration is a feature of life in many such countries (19, 20).

**Fig. 1:**
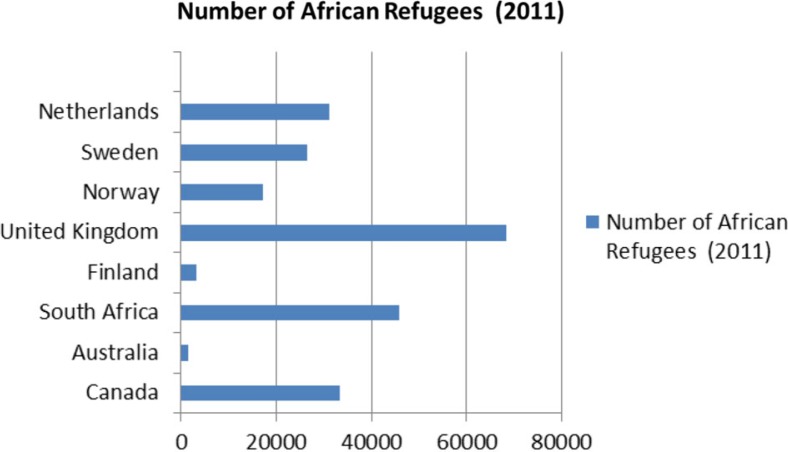
Number of African refugees in different countries (18).

The everyday experience of trying to provide mental health services in linguistically diverse low-income contexts can be very difficult. One of the authors of this article, for example (JT), worked as one of three social workers at a large mental health referral hospital in Uganda, which has 43 languages, and took service users from a variety of contexts. Box 1 summarises the challenges he faced in trying to provide mental health services to a linguistically diverse service user population.

Box 1 **Difficulties faced by a social worker providing mental health services in a hospital in Uganda**

1. Inability to communicate with service users.
2. Inability to assess large numbers of referrals of children with speech impairments through not understanding their languages.
3. No sign language services.
4. No linguistic access for service users from neighbouring countries such as Rwanda who spoke either Kinyarwanda or French.
5. Difficulty in educating service users about their psychiatric diagnoses. It was, for instance, impossible to find local equivalents for terms such as schizophrenia and bipolar disorder in my first language.
6. It was impossible to translate the emotionally loaded and culturally nuanced local idioms of distress into a psychiatric diagnosis.
7. Referral for specialist services was not possible in the absence of specialists speaking local languages, and in the absence of interpreters.
8. Discussion of confidential matters was impossible when family members acted as interpreters.

## Some implications and possible ways forward

The global mental health movement has gone a long way in developing methods of intervention which are appropriate to local contexts, and which make use of local resources (including local cultural and linguistic resources). As global mental health ‘comes of age’, as has been suggested, however, and interventions need to be scaled up, innovative ways of dealing with linguistic and cultural diversity need to be developed (21). Much can be learned from the literature on interpreting in mental health in high-income countries, but interpreter services are expensive, and the models of wealthier countries cannot be imported.

We suggest as a first step to tackling the complex question of language issues in mental health services in low- and middle-income countries, it would be useful for those who are working innovatively to develop locally delivered mental health programmes to explore and report on issues of language and how these have been addressed. We need to know more about local challenges, but also about local solutions which seem to work, and for this we need far more information from the field than is currently available. It is clearly not sufficient to think about translating instruments into one or two local languages. We need to develop knowledge and skills to cope with linguistic complexity, and we need to gain this knowledge from on the ground experiences in a range of contexts.

Our own group is experimenting with low-cost lay interpreter services in local hospitals and we will be reporting on this work in detail in due course. In a pilot study in institutional mental health care in South Africa, we have developed a brief training programme for lay interpreters. At this stage, we have considerable anecdotal evidence that having this small cadre of trained and supervised interpreters does increase access, but larger scale implementation and evaluation in a wider range of contexts is essential. One of the challenges associated with increasing language access to services is that services themselves need to adapt to a situation in which health care users may become more vocal in interaction with services, so all interventions have to be explored with respect their organisational dimensions as well with respect to the purely linguistic dimensions. A major challenge will be in bringing these innovations to scale. Our intervention will be nowhere near sufficient to address the language issues in the global mental health field. A diversity of reports and suggestions is required. The global mental health field needs a concerted and collaborative focus on language as a key issue. Though local and contextual solutions are important, there will in all likelihood be important lessons to be learned through sharing and collaboration.
